# Microneedle-Based Technologies for Long-Acting Transdermal Drug Delivery in Wearable Devices

**DOI:** 10.3390/s26010239

**Published:** 2025-12-30

**Authors:** Jiaxin Luo, Yinqi Dai, Xin Cheng, Zifeng Wang, Zhigang Zhu

**Affiliations:** School of Health Science and Engineering, University of Shanghai for Science and Technology, Shanghai 200093, China; 232322239@st.usst.edu.cn (J.L.); yqdaiwy@163.com (Y.D.); 254302320@st.usst.edu.cn (X.C.); zfwang@usst.edu.cn (Z.W.)

**Keywords:** microneedles, transdermal drug delivery, long-acting release, controlled release, biodegradable materials

## Abstract

This review systematically outlines recent advances in long-acting microneedle-based transdermal drug delivery systems. It begins by introducing the fundamental principles of microneedles (MNs) as a minimally invasive technology and categorizes them by delivery mechanism into solid, coated, dissolving, hollow, hydrogel-forming, and biodegradable types. The review then discusses the design strategies and material platforms engineered for sustained drug release. A key focus is on biodegradable synthetic polymers, such as polylactic acid (PLA), poly (lactic-co-glycolic acid) (PLGA), and polycaprolactone (PCL), and natural polymers like silk fibroin (SF) and chitosan (CS), which enable prolonged drug release through their tunable degradation rates. Furthermore, it describes the incorporation of advanced drug carriers, including liposomes and polymeric nanoparticles/microparticles, into MNs to further extend release duration and enhance drug-loading capacity. Finally, the major challenges for clinical translation are addressed, including ensuring batch-to-batch consistency in manufacturing, maintaining sterility, and the necessity for more comprehensive validation of long-term in vivo efficacy and safety.

## 1. Introduction

Effective management of chronic conditions, including skin infections, wound healing, and antiviral therapies for diseases like HIV and Hepatitis B, requires drug delivery systems capable of sustained release [1]. For example, in chronic skin infections, pathogenic bacteria can penetrate the skin layer and form biofilms in the underlying tissue. These biofilms are 1000 times more resistant to antibiotics than free bacteria. Maintaining effective local drug concentration at the infection site can greatly improve the treatment effect. However, traditional topical preparations, such as ointments and gels, have low efficiency in delivering drugs to the stratum corneum, which is the outer barrier of the skin, and cannot maintain a continuous treatment level [2]. To solve the limitations, an MN-based transdermal drug delivery system was constructed. Compared with oral administration (affected by first-pass metabolism) and injection administration (invasive and painful), microneedles (MNs) provide a friendly choice for patients. They bypass the stratum corneum (a key limitation of traditional transdermal drug delivery) to ensure reliable drug delivery [3].

MNs belong to a new type of minimally invasive transdermal drug delivery system. Their diameter is usually 100–1000 μm. It is designed to penetrate the stratum corneum of the skin surface and not reach the deep dermis containing nerves. It can achieve effective drug administration with minimal invasion and pain [4]. Compared with traditional transdermal patches, MNs can increase the drug permeability by 10–100 times [5]. It greatly promotes the delivery of drugs with a wide range of molecular weights, which are usually difficult to effectively penetrate the skin barrier [6]. The multi-functionality of materials enables MNs to be designed for drug delivery and functional applications, enhancing the flexibility of transdermal drug delivery. The application of MNs is relatively simple, and patients can manage it by themselves, which may reduce the medical burden. This review elaborates on the classification of MNs, summarizes the progress of long-acting MNs, describes the types of sustained-release MNs, and analyzes the design and application.

## 2. Classification and Preparation of MNs

### 2.1. Categorized by Drug Delivery Mechanism

**Figure 1:**
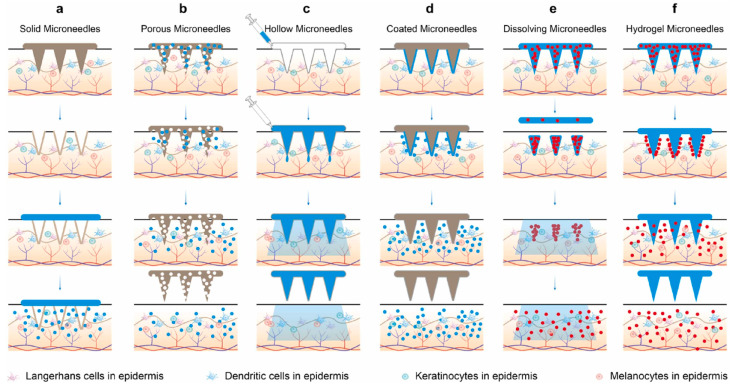
The drug delivery mechanisms of six types of MN. (**a**) Solid MNs, (**b**) Porous MNs, (**c**) Hollow MNs, (**d**) Coated MNs, (**e**) Dissolving MNs, and (**f**) Hydrogel MNs. Reproduced with permission from Zhai et al. (2025) [7] © 2025 Elsevier B.V.

MNs can be classified by drug delivery mechanism into several types: solid (Figure 1a), porous (Figure 1b), hollow (Figure 1c), coated (Figure 1d), dissolving (Figure 1e), hydrogel (Figure 1f), and biodegradable MNs.

Solid MNs are typically made from metals (e.g., titanium, stainless steel), silicon, glass, or polymers like PLA [8] and polystyrene (PS). These are solid, drug-free structures used primarily for skin pretreatment. They first pierce the stratum corneum to create microchannels. After the MN array is removed, a topical drug is applied to the skin surface, allowing it to diffuse through these channels into the dermis. This approach is often used to enhance transdermal patches, significantly improving the skin permeability of small-molecule drugs, such as local anesthetics and antivirals, by bypassing the stratum corneum barrier [9].

Porous MNs have small pores for loading drugs. After being inserted into the skin, the drug is released by diffusion [10]. A key advantage of this design is its high efficacy in delivering high-molecular-weight drugs. And it has little impact on drug activity, which is beneficial for sensitive bioactive substances. However, the pores also bring engineering difficulties, and the balance between drug delivery and MN mechanical strength is needed.

Dissolving MNs are composed of water-soluble materials with drug matrices [11,12]. Different from solid MNs, they stay on the skin after insertion. The needle tips absorb interstitial fluid, making the matrix dissolve and release drugs. For example, carboxymethyl cellulose (CMC)-based MNs containing silver nanoparticles dissolve rapidly after insertion, releasing silver ions that inhibit both Escherichia coli and Staphylococcus aureus. And no cytotoxicity was observed in cells treated with these MNs [13].

Hollow MNs feature a central bore that maintains a continuous channel for drug delivery after skin insertion, enabling efficient and sustained administration. It has advantages in rapid drug delivery. Studies have shown that T4 phages can be delivered into the pig skin model, and the phages can still be detected in the rat serum within thirty minutes, which opens a new way for treating Escherichia coli infection [14]. However, drug release via passive diffusion is limited, often requiring external pressure (e.g., from a syringe or pump) to achieve delivery.

Coated MNs are solid MNs that form drug coatings (with thickness ranging from nanometers to micrometers) on the surfaces of polymers, metals, and silicon [15] by means of dip-coating, spray-coating, or laser deposition. After penetrating the stratum corneum, the coating meets the interstitial fluid and dissolves, releasing the drug into the dermis. Coated MNs offer a rapid onset, making them advantageous for low-dose drugs [16]. However, the limited drug-carrying capacity restricts the dosage administered, and sustained release is difficult to achieve.

Hydrogel-forming MNs are a new type of MNs composed of cross-linked three-dimensional polymer networks such as hyaluronic acid methacrylate (HAMA) or polyvinyl alcohol (PVA) [17]. The drug is loaded in this hydrogel matrix. The MNs have strong hydrophilicity. After being inserted into the skin, they swell by absorbing interstitial fluid rather than dissolving. This swelling forms a drug reservoir in the skin, and the drug diffuses out through the swollen network, thus achieving sustained release. The release curve is affected by the swelling rate and crosslinking density and can also be designed for stimulus-responsive drug delivery. Wang et al. developed ROS-responsive swelling MNs for the treatment of acne. The high concentration of ROS in the acne site will degrade the polymer network and accelerate the release of clindamycin to carry out controlled release treatment [18].

Finally, biodegradable MNs need to rely on the gradual degradation of the needle-like material to form an in situ drug reservoir for continuous release. They are made of super biocompatible and degradable materials, such as PLGA, PLA, CS, and PCL [19]. Their advantage lies in being able to deliver drugs for a long time, and the drug reservoir remains in the body for a long time due to the slow degradation. This makes them suitable for chronic diseases, such as fungal infections and long-term antiviral treatment [20]. For example, Yang et al. reported that the fluconazole-loaded polylactic-co-glycolic acid copolymer MNs degrade in vitro in about 3 days, can continuously release drugs in vivo for up to 2 weeks, have a drug concentration in the skin that is about 6.5 times that of the traditional patch, and greatly reduce the frequency of administration [21].

### 2.2. Categorized by Material

MNs can also be categorized by their constituent materials, such as polymers, metals, natural materials, glass/silicon, and ceramics. The choice of material directly determines an MN’s mechanical properties, biocompatibility, drug release profile, and suitability for specific applications.

**Figure 2:**
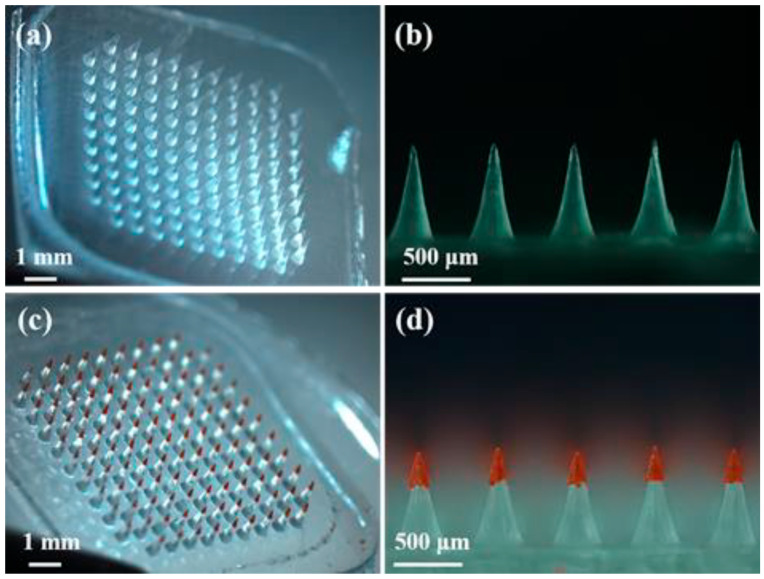
Optical images of PLGA-based MN. (**a**) Stereomicroscopic image of water-soluble MNs. (**b**) Side view of water-soluble MNs. (**c**) Stereomicroscopic image of FR-loaded IPMNs. (**d**) Side view of FR-loaded IPMNs. Reproduced from Zhao et al. (2020) [29], under the terms of the Creative Commons Attribution License.

Polymer-based MNs (MNs) are widely used (As shown in Figure 2a–d). Common materials include PVP, CMC, hyaluronic acid (HA), PLGA, PCL, PLA, and CS [22]. The advantages are that they have good biocompatibility and can also adjust properties to meet the needs of specific treatments. Due to the large variety of polymers, MNs have functional applications. For example, PVP, CMC, and HA can be made into fast-dissolving MNs to achieve rapid drug delivery [23,24]. PLGA, PLA, and PCL are suitable for the preparation of sustained-release long-acting MNs [25,26]. PVA and CS can form hydrogel MNs for multifunctional delivery [27,28]. Due to their material versatility, polymer-based MNs can address the vast majority of transdermal delivery scenarios and remain among the most promising research directions.

Metal MNs are made of materials such as stainless steel, titanium, and gold [30]. They have high strength and hardness and can penetrate the skin. This kind of metal MN is long-term stable and reusable, which may reduce costs. However, their solid nature makes direct drug loading challenging. They are primarily used either to pre-treat the skin for subsequent application of a drug patch or to engineer hollow MNs as conduits for sustained drug delivery [31]. While their mechanical advantages are apparent, limitations in drug incorporation have hindered transformative advances in drug delivery applications.

MNs based on natural materials such as gelatin and maltose have attracted increasing attention. These materials are from nature and often show good biocompatibility. This property makes them attractive in antibacterial MN applications [32]. They also have unique advantages in specific scenarios; for example, maltose MNs used in the gastrointestinal environment can avoid immune responses and can safely deliver drugs [33]. Gelatin is valued because of its good biocompatibility and easy accessibility and is a common choice, as demonstrated by Wang et al. [34]. Gelatin-based hydrogel MNs are used for curcumin delivery to treat infected wounds. [35] Lee et al. developed porous silk fibroin MNs for drug delivery to vascular sites. Natural materials have the advantage of biocompatibility in drug delivery.

Glass and silicon nanoneedles with quartz glass or monocrystalline silicon as the main materials have relatively good biocompatibility. The size and sharpness of the needles can be precisely controlled during manufacturing. The surface can be easily modified with functional components such as antibodies or sensors. However, due to material limitations and high costs, applications are mostly only in basic research laboratories of transdermal drug delivery mechanisms [36].

Ceramic MNs are made of hydroxyapatite, zinc oxide, and titanium dioxide. They have high biocompatibility, hardness, and heat resistance. For example, zinc oxide can release zinc ions to inhibit microorganisms. These characteristics make them suitable as antibacterial systems and can also be used for local drug delivery near bones [37].

### 2.3. Preparation of Microneedles

As an efficient transdermal drug delivery platform, the fabrication of microneedles (MNs) has evolved with advances in materials science and micro-manufacturing. The chosen process depends on the MN type (e.g., coated, dissolving, porous, solid), material properties, and the intended application. A key objective is to balance mechanical strength, biocompatibility, drug-loading efficiency, and scalable production. Core technical considerations include mold fabrication, material forming, and structural optimization. Polydimethylsiloxane (PDMS) molds are a central tool due to their high replication fidelity and reusability.

For metal or specific polymer MNs, laser-based techniques are often employed [38]. One method involves laser-cutting a two-dimensional shape from a CAD design, which is then bent into form, followed by electropolishing to achieve a smooth surface. Photolithography is fundamental for creating MNs with complex architectures [39]. This process uses a photomask to transfer a pattern onto a light-sensitive polymer, followed by curing and etching to produce solid or hollow silicon or polymer MNs. Etching methods include dry etching (highly precise and anisotropic) for high-fidelity tips and wet etching (faster and lower cost) for bulk production [40,41]. In a molding process centered on a flexible PDMS mold, a master structure is first created, typically from SU-8 photoresist or machined metal (e.g., nickel, tungsten, titanium) [42]. The PDMS prepolymer and curing agent (often in a 10:1 ratio) are mixed, degassed, poured over the master, and cured at ~60 °C for 1–2 h. Upon cooling and demolding, a complementary PDMS MN mold is produced. This PDMS mold can then be used with centrifugal filling, vacuum assistance, or UV curing to fabricate coated, dissolving, or porous MNs. The process is relatively simple, and the cleaned PDMS mold is reusable, making it well-suited for lab-scale prototyping and small-batch production. Common materials include water-soluble or biodegradable polymers like PVA, PVP, and hyaluronic acid [43,44]. Three-dimensional (3D) printing, or additive manufacturing, enables the creation of MNs with intricate internal structures that are difficult to achieve with traditional methods, offering advantages in precision and rapid prototyping [45]. For coated MNs, various techniques such as dip-coating, spray-coating, air-jet drying, inkjet printing, and electrohydrodynamic atomization can be employed to efficiently apply metallic or polymeric coatings to MN substrates [46,47].

## 3. MNs as a Strategy for Long-Acting Drug Delivery

**Figure 3:**
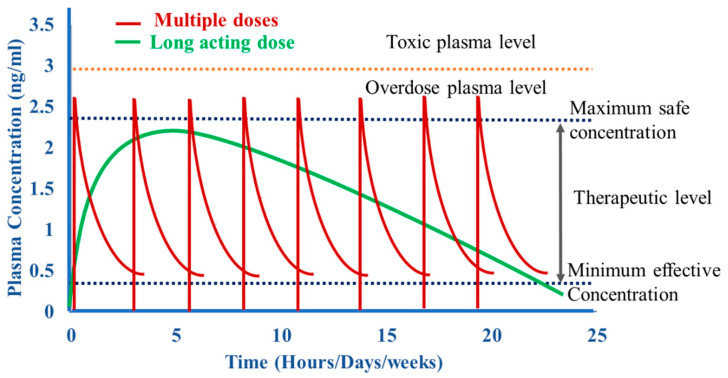
Pharmacokinetics of multiple dosing and sustained-release drugs. Reproduced with permission from Vora et al. (2021) [48] © 2021 Elsevier B.V.

MNs are the focus of transdermal drug delivery research, and they are gradually receiving attention in the application of long-acting preparations. The traditional frequent administration regimen leads to large peak/trough fluctuations in plasma drug concentration, which compromises the drug efficacy and utilization rate. The long-acting system is shown in Figure 3 [48]. It wants to overcome this problem by maintaining stable blood drug concentration and improving treatment efficiency. Polymer-based MN systems are very promising in long-acting drug delivery. We designed and utilized the biodegradability of polymers to fabricate structures that can release drugs for a long time. PLGA copolymer MNs have sufficient mechanical strength to penetrate the skin. The degradation rate of PLGA can be adjusted by the ratio of lactic acid/glycolic acid monomers. Controlled degradation helps to form a local drug reservoir in the skin, which can be used for long-acting MN patches [49]. Another strategy is to use a three-dimensional hydrogel network. The drug release rate can be adjusted by the crosslinking density of the hydrogel; environmental response mechanisms can be added to achieve intelligent on-demand drug release [50]. The integration of drug-loaded nanoparticles or microparticles is an important direction. The carrier encapsulates the drug for controlled release so that the time for maintaining the therapeutic concentration is longer than that of the traditional system. MNs are efficient tools for delivering carriers across the skin barrier [51]. More complex and customized release processes can be achieved by combining methods such as polymer degradation, hydrogel diffusion, and particle-based delivery.

The fabrication of long-acting drug delivery microneedles primarily utilizes polymers with good biocompatibility and tunable mechanical properties. Biodegradable synthetic polymers, such as PLGA and PCL, serve as core matrix materials due to their precisely adjustable degradation period (ranging from weeks to months), enabling sustained release. Natural polymers and their derivatives, including silk fibroin and chitosan, are often employed to construct sustained-release or stimulus-responsive microneedles, owing to their excellent bioactivity and modifiability. These materials are typically processed via techniques like micro-molding into biodegradable matrices, particle-loaded composite structures, or core–shell hierarchical systems. This integrated approach simultaneously addresses the key requirements of high drug-loading capacity, controlled release, and minimally invasive delivery. Subsequently, this paper systematically reviews recent advances in long-acting drug delivery microneedles. We summarize and analyze their design strategies, drug release mechanisms, and performance efficacy, with the aim of clarifying the current developments and future trends in this technology.

### 3.1. PLA/PLGA MNs

**Figure 4:**
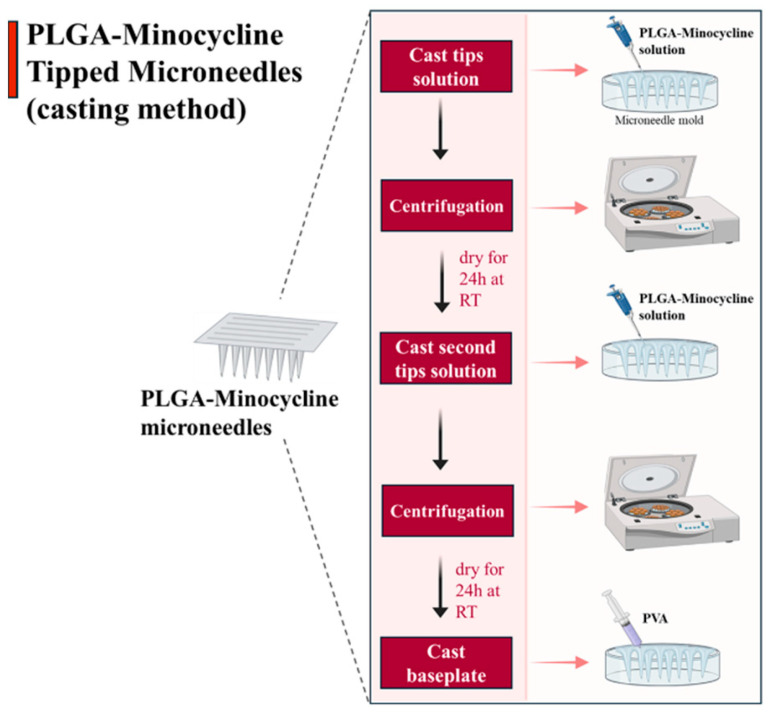
Preparation of MNs by the centrifugal casting method. Reproduced from Abu et al. (2025) [55], under the terms of the Creative Commons Attribution License.

PLA and PLGA are commonly used synthetic biodegradable polymers. They have good biocompatibility and can regulate degradation, showing good performance in biomedical applications [52]. 3D printing is often used to fabricate MNs, and PLA is particularly suitable for rapidly and precisely fabricating MN arrays [25]. However, pure PLA has low thermal stability and is relatively brittle during 3D printing, and it is easy to deform when fabricating MNs. Adding chitin to PLA can solve these problems. Chitin can make the material have better toughness, and its hydrogen bonding ability is helpful for drug loading [53]. Solution casting is a commonly used MN preparation technology, which can replace 3D printing [54,55]. In this process, the polymer is dissolved in the solvent, and the mold is filled by centrifugal force to make MNs with sufficient needle tip length, as shown in Figure 4.

To achieve more controlled drug release, researchers have added trehalose to PLA MNs, which significantly enhances drug delivery efficiency, enabling the release of both hydrophobic and hydrophilic drugs within 30 min [56]. PLGA is a random copolymer synthesized from lactic acid (LA) and glycolic acid (GA) monomers. A key characteristic of PLGA is that its degradation rate can be modulated by varying the LA-to-GA ratio. For instance, one study reported that when the LA:GA ratio was 75:25, the cumulative drug release measured in vitro over 7 days decreased by approximately 6% compared to a ratio of 50:50 [57]. PDMS mold centrifugal casting is usually used to prepare PLGA MNs, where the drug is at the tip position, and a backing layer is formed using materials such as PVP or PVA [58]. It should be noted that in addition to being controlled by the monomer ratio, PLGA MNs’ drug release can also be adjusted by blending with hydrophilic polymers. Apoorva et al. added PVA into PLGA MNs and found that 5% w/w PVA could most effectively promote drug release and improve drug utilization [59]. Zhao et al. incorporated trehalose as a porogen into PLGA microneedles to accelerate drug release and enhance the cumulative release rate. The addition of 33.3% trehalose increased the drug release over 21 days from 60% to 76%, compared to microneedles without trehalose. Furthermore, the authors conducted an in vivo degradation study using implantable PLGA microneedles (IPMNs) loaded with fluorescent rhodamine (FR), which were inserted into rat skin and monitored for 3 weeks. The degradation process was visualized via histological sectioning and examined under bright-field and fluorescence microscopy. A comparison between the group without trehalose and the group with 33.3% trehalose revealed that the trehalose-added PLGA microneedles degraded faster due to their more porous structure and increased surface area, with an estimated complete degradation time of 4–6 weeks, versus 6–8 weeks for those without trehalose [29]. In addition, to meet the needs of the initial burst release of some drugs, the combination of coating and degradable MNs can be adopted. Apply the drug on the surface of PLGA MNs for immediate release, and the PLGA matrix encapsulates the drug for long-term sustained release [60]. Prepare MNs using PLGA and hydrophilic material (such as PVP or HA) to achieve biphasic release. The hydrophilic part helps the initial burst release, and PLGA is used to maintain the extended release [61].

To make long-acting MNs play their role on the skin for a long time, designing a detachable structure is effective. A microneedle system featuring a hydrogel backing layer and a PVP separation layer was developed by Ke Peng et al. This design offers multiple advantages: the hydrogel backing is water-insoluble and non-adhesive to the skin, allowing for easy removal after application. The system enables rapid implantation within one minute, improving user comfort. Meanwhile, the needle tips, fabricated from PLGA with an LA:GA ratio of 75:25, are loaded with amphotericin B and facilitate drug release over one week [62]. Chen et al. developed a bilayer microneedle system comprising PLGA needle tips loaded with curcumin and a hyaluronic acid (HA) base layer containing gallic acid. This design enables coordinated drug release: the HA layer dissolves within five minutes to rapidly deliver its payload, while the PLGA needle tips (with an LA:GA ratio of 75:25) remain embedded in the dermis to provide sustained drug release for up to two months [63].

### 3.2. PCL MNs

**Figure 5:**
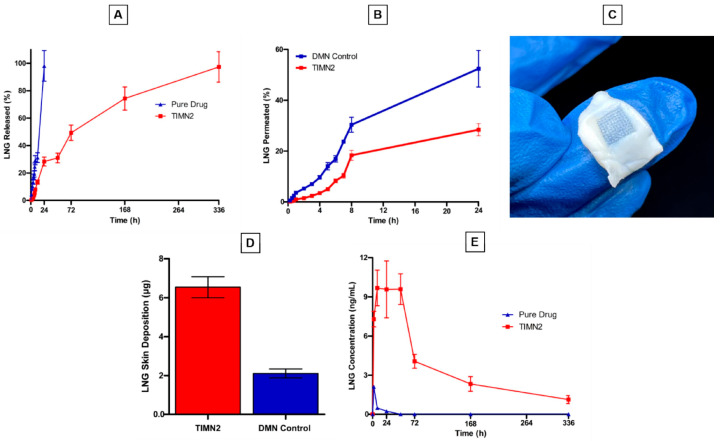
In vitro and ex vivo characterization of levonorgestrel (LNG)-loaded PCL MNs (TIMN2). (**A**) Cumulative drug release profile of TIMN2 compared to pure LNG. (**B**) Ex vivo skin permeation profile of TIMN2 compared to a control group without PCL (DMN). (**C**) Representative images showing skin permeation following the ex vivo test. (**D**) Quantification of LNG deposition in the skin after application of TIMN2 versus the DMN control. (**E**) Mean plasma concentration-time curve of TIMN2 compared to an oral suspension. Reproduced with permission from Permana et al. (2025) [66] © 2025 Elsevier B.V.

PCL is a synthetic polyester with semicrystalline properties and biodegradability [64]. PCL exhibits good biocompatibility but poor water solubility, making it suitable for fabricating long-acting microneedles. In a study on the transdermal delivery of allopurinol for acute hyperuricemia in mice, a microneedle system employing PCL and PVP demonstrated no significant inflammatory response and lower systemic side effects compared to oral administration. However, microneedles composed solely of PCL are excessively hydrophobic, which can impede drug release [65]. Researchers have incorporated additional polymers into PCL to modulate the drug release profile. For instance, Andi Dian Permana et al. developed a biphasic-release MN patch composed of PCL and PVP for the sustained delivery of levonorgestrel. PVP dissolves rapidly, with an initial burst release of 28% within 24 h, and then the sustained release controlled by PCL can be maintained for 14 days (Figure 5A). Thus, the plasma drug concentration remains above the therapeutic level within 14 days (Figure 5E). In vitro skin penetration studies demonstrate that PCL significantly slows the release rate and prolongs the release period compared to MNs without PCL (Figure 5B). Moreover, drug deposition in the dermis after 24 h was substantially higher in the PCL group (Figure 5D) [66]. Nur et al. developed a three-layer dissolving MN system for the treatment of long-term Alzheimer’s disease, which contains rivastigmine. The needle tip is made of PCL and PVP when inserted. When the water-soluble PVA/PVP backing layer dissolves, the needle tip is designed to remain in the skin. This structure can achieve sustained drug release for 168 h [67]. Polyethylene glycol (PEG) is another material used in MNs together with PCL to promote drug release [68]. The drug release rate can be modulated by varying the concentration of polyethylene glycol (PEG). Research indicates that a higher PEG content effectively accelerates release. For instance, Bader et al. investigated the effect of incorporating 1% and 0.5% PEG into a 5% PCL matrix. Their results showed that the formulation with 1% PEG released 60% of its drug payload within 24 h, compared to only 29% for the 0.5% PEG formulation. Following this initial phase, both formulations exhibited sustained release over a period of 20 days [69]. In addition to the physical blending method, the release of drugs can also be regulated by chemically modifying PCL. For example, Anna et al. prepared a photocrosslinked acrylate-terminated urethane-based polymer (AUP-PCL) using PCL. When unmodified PCL interacts with cortisol, the drug release can last for 2 days, while AUP-PCL extends the drug release time to 3 weeks [70].

### 3.3. Silk Fibroin (SF) MNs

SF, a natural polymer, has garnered significant interest as a matrix material for MNs due to its excellent biocompatibility, tunable biodegradability, favorable mechanical properties, and ease of processing [71]. SF MNs are prepared by a mild all-aqueous phase process, without the use of organic solvents, which can maintain drug activity and remain stable in the SF matrix. In addition to pure SF, functional components are added to improve performance. For example, forming an interpenetrating network (IPN) hydrogel with HAMA to increase mechanical strength and alleviate the brittleness of natural SF [72]; adding proline to adjust the protein crystal structure, thereby affecting drug release [73]; and blending with CS to increase mucosal adhesion and prevent MN detachment [74].

**Figure 6:**
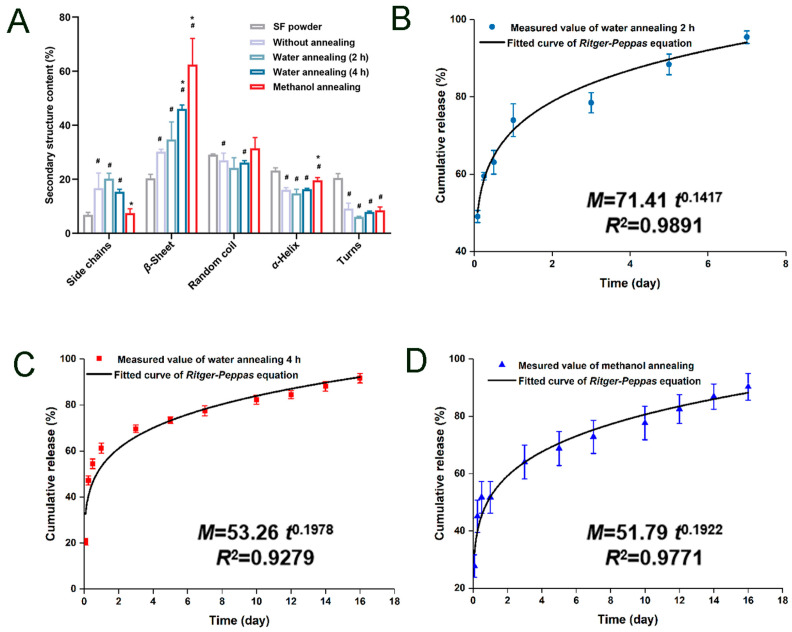
Influence of SF Secondary Structure on Drug Release Performance. (**A**) Secondary structure content of MN patches with different annealing condition. Data are mean ± SD, *n* = 3; *p* < 0.05 vs. SF power; * *p* < 0.05 vs. Without annealing. (**B**–**D**) Equation fitting for release profiles of MN patch water annealing for 2 h, 4 h and methanol annealing for 0.5 h. ^#^ Reproduced from Yang et al. (2023) [80], under the terms of the Creative Commons Attribution License.

In the secondary structure of SF, the content of β-sheet is the key to the mechanical properties of MNs and drug release. Techniques such as ethanol soaking [75], methanol vapor treatment [76], water vapor annealing [77], or the addition of specific reagents (such as proline [78]) will change the conformation from random coil to β-sheet. As shown in Figure 6, this transformation directly affects the drug release curve. For example, Yan et al. used methanol vapor treatment to increase the content of β-sheet and prepared MNs that can continuously release drugs in vitro for 7 days [79]. Similarly, Li et al. fabricated silk fibroin (SF) microneedles loaded with recombinant human growth factor using a water vapor annealing method. Structural analysis revealed that the β-sheet content of SF was 34% after 2 h of water vapor annealing and increased to 46% after 4 h. In contrast, methanol annealing for just 0.5 h resulted in a significantly higher β-sheet content of 62% (Figure 6A). Importantly, the drug release profile differed accordingly: microneedles annealed by water vapor for 2 h sustained release for 7 days, whereas both those annealed by water vapor for 4 h and those annealed by methanol for 0.5 h extended the release duration to 14 days, with no significant difference observed between these two longer-duration groups. The study further reported that the release mechanism in all cases was primarily governed by Fickian diffusion, as illustrated in Figure 6B–D [80]. The incorporation of proline induces the formation of Silk I crystalline structures within silk fibroin (SF), which contributes to controlled drug release. Notably, varying the proline content significantly alters the crystallinity of SF microneedles, thereby yielding distinct drug release profiles. Wang et al. investigated the effect of different proline-to-SF mass ratios on release kinetics. Microneedles with proline/SF mass ratios of 0.5:10, 1:10, and 3:10 exhibited linear release phases lasting 8–36, 8–48, and 8–60 h, respectively, with corresponding cumulative release rates of 81.6%, 80.8%, and 67.9% [81]. Isopropanol has also been shown to gel SF solutions by promoting β-sheet formation [82]. Higher β-sheet content can enhance mechanical strength, reduce swelling rate, and slow down the drug diffusion rate, and the release time can be adjusted from several hours to several months.

The excellent biocompatibility of SF enables highly flexible drug loading, accommodating a wide range of molecules, including proteins (e.g., insulin, growth hormone, vaccine antigens), small molecules (e.g., melatonin, triptorelin, prednisone), nucleic acids, and exosomes [83,84]. The cytocompatibility of silk fibroin (SF) microneedles (MNs) was evaluated by Lin et al. MC3T3 cells cultured with extracts from the MNs showed robust proliferation without signs of cytotoxicity. Furthermore, after 24 h of culture directly on SF substrates, the MC3T3 cells had spread across the surface, demonstrating favorable biocompatibility [77]. In a separate study, Wang et al. fabricated MNs from a blend of SF and chitosan (CS). Biocompatibility was assessed using L929 cells via live/dead staining and CCK-8 assays, revealing a cell viability of 122.1%, which was attributed to the excellent biocompatibility of the SF and CS fibers. In an in vivo diabetic wound model, treatment with these composite MNs reduced inflammatory cell infiltration, promoted the polarization of macrophages from the M1 to the M2 phenotype, and accelerated wound healing without observed toxicity, outperforming the control group [74]. Diverse release mechanisms have been engineered for specific therapeutic needs. Guan et al. developed a photocrosslinked silk fibroin (SF)-based hydrogel microneedle patch for diabetic wound healing. The crosslinking process enhanced the mechanical strength and stability of the patch. Experiments demonstrated a compressive fracture force of 0.42 N per needle, which is two to three times greater than that of pure SF microneedles, along with excellent swelling properties. The patch was designed to deliver epigallocatechin gallate (EGCG), a multifunctional agent. In vitro tests confirmed its effective inhibition of Staphylococcus aureus and Escherichia coli, as well as a high DPPH free radical scavenging rate of 90.2%. In a diabetic mouse model with infected wounds, in vivo results showed that the treatment group using these microneedles achieved a wound healing rate of 96.9%, significantly higher than the 87.1% observed in the commercial dressing control group. Furthermore, the treatment promoted organized collagen deposition and accelerated the shift of macrophages from the pro-inflammatory M1 phenotype toward the reparative M2 phenotype [85]. Jia et al. developed a hollow SF MN system for sustained transdermal delivery of liraglutide, leveraging the hollow design’s high drug-loading capacity to provide continuous delivery for 24 h and reduce dosing frequency [86]. Furthermore, the integration of smart materials (e.g., thermo- or pH-responsive) enables on-demand, triggered drug release for specific application scenarios [87,88].

### 3.4. CS MNs

CS, an abundant and biocompatible natural polysaccharide, has become a core material for novel transdermal MNs due to its biodegradability, excellent film-forming ability, and easily modifiable functional groups [89]. With cationic charges, it can have electrostatic interactions with negatively charged drugs or proteins, thus increasing the drug loading [90]. Chemical modification of chitosan can improve the drug loading, mechanical properties, and release characteristics. For example, water-soluble chitosan is obtained by mild acid hydrolysis with trifluoroacetic acid, and this chitosan can be processed at neutral pH and room temperature to load pH-sensitive biological agents [91]. Dai et al. introduced methacryloyl groups to prepare methacrylated chitosan (CSMA) so that it forms a photocrosslinked hydrogel network with good mechanical strength (up to 0.7 N per needle), and this network had stronger insertion force than the skin. It can cope with harsh conditions such as psoriasis skin. In the psoriasis mouse model, the CSMA hydrogel MNs loaded with methotrexate and nicotinamide successfully penetrated the skin, carried out continuous release, significantly inhibited skin thickening and inflammation, and almost solved the lesions within 9 days [92]. Wei et al. used carboxymethyl chitosan (CMCS) cross-linked with oxidized branched starch through Schiff base bonds to prepare hydrogel MNs with good swelling and degradability [93].

**Figure 7:**
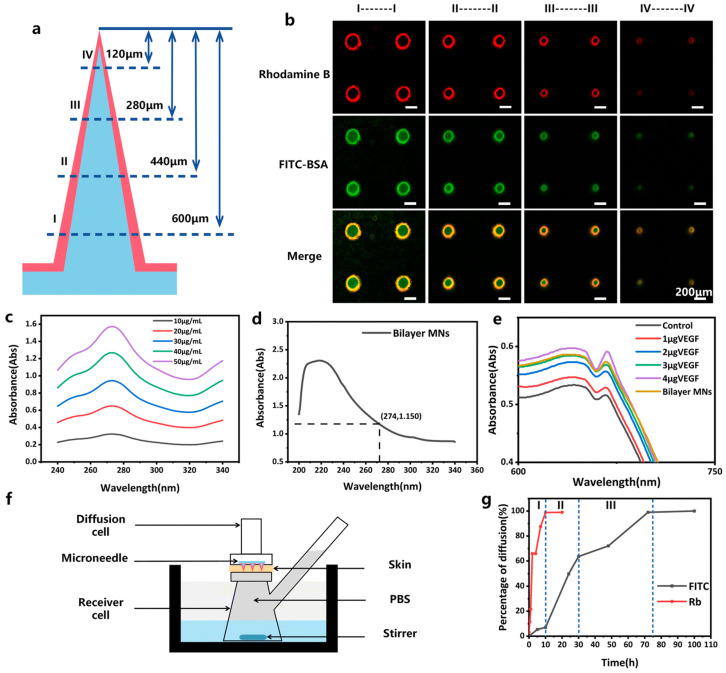
CS/HA Bilayer MNs. (**a**) Schematic cross-sections of the CS/HA bilayer MN at 600 (I), 440 (II), 280 (III), and 120 (IV) μm from the needle tip; (**b**) Representative confocal microscopy cross-sectional images corresponding to the levels shown in (**a**). Green fluorescence indicates FITC-BSA (located in the core), and red fluorescence indicates Rhodamine B (located in the shell). Scale bar: 200 μm; (**c**) Absorption spectra of PBS solutions spiked with doxorubicin at concentrations of 10, 20, 30, 40, and 50 μg mL^−1^; (**d**) Absorption spectrum of a PBS solution containing dissolved CS/HA bilayer MNs, showing an absorbance value of 1.15 at 274 nm; (**e**) Absorption spectra of Coomassie Brilliant Blue solutions after treatment with 1, 2, 3, and 4 μg of vascular endothelial growth factor (VEGF) or with the CS/HA bilayer MNs; (**f**) Schematic diagram of the in vitro drug release mechanism; (**g**) Drug release profiles of FITC-BSA and Rhodamine B from the CS/HA bilayer MNs in SD rat skin. Reproduced from Zhong et al. (2025) [97], under the terms of the Creative Commons Attribution License.

CS MNs have great design flexibility and diverse functions in drug delivery and disease treatment. Chen et al. fabricated microneedles with chitosan (CS) needle tips loaded with ovalbumin (OVA) and a rapidly dissolving PVA/PVP backing layer to enable efficient intradermal implantation. Following microneedle insertion, visible erythema (skin redness) was observed after 15 min, but this erythema lasted only a few hours, with the skin returning to its normal appearance within 1–3 days. The microchannels created by the microneedles began to close within 15 min and were completely closed by 6 h, indicating that the microneedle-induced skin irritation was mild and reversible. Histological analysis confirmed the absence of significant inflammation. The CS microneedles subsequently underwent gradual degradation in the skin, remaining visible though reduced in size over 28 days, demonstrating favorable degradation properties. Furthermore, they functioned as an intradermal depot, sustaining antigen release for 3–4 weeks [94]. Chiu et al. designed a bilayer microneedle system consisting of a hyaluronic acid (HA) needle tip and a chitosan (CS) base. This design facilitates biphasic drug release kinetics—an initial burst release followed by sustained release—leveraging the rapid dissolution of HA and the prolonged release properties of CS. In their study, ovalbumin (OVA) was fluorescently labeled and separately loaded into the HA tip and the CS base. Over a 7-day period, the OVA encapsulated in the HA component was completely released, whereas only 35% of the OVA loaded in the CS base was released [95]. Thiolated chitosan (TCS), modified with thiol groups, exhibits enhanced mucoadhesive properties and improved drug permeation capability. Ahmad et al. demonstrated that when tacrolimus was delivered using TCS microneedles, 92% of the drug was released within 24 h. In contrast, a conventional tacrolimus ointment released only 53% over 48 h. The TCS microneedle system thus showed superior transdermal delivery efficiency and bioavailability [96]. Zhong et al. used spray drying to make double-layer MNs, with the core being CS and the shell being HA, for multi-drug-controlled release (Figure 7a) [97]. Confocal microscopy confirmed the successful formation of the bilayer, with FTIC-BSA in the core position and rhodamine B in the outer shell part (Figure 7b). This system can enable the sequential release of VEGF and doxorubicin (Figure 7g). The MNs combined with angiogenic and antibacterial effects completely closed the full-thickness skin defect of mice within 9 days, significantly accelerating the wound healing process. Wang et al. incorporated phenylboronic acid (PBA)-modified chitosan (CS) particles into a PVA/PVP hydrogel microneedle system. These PBA-CS particles confer glucose-responsive properties to the microneedles. Experimental data showed that when the glucose concentration increased from 0 to 400 mg/mL, the drug release efficiency was enhanced by approximately 10% [98]. 

### 3.5. Polymeric Microparticles and Nanoparticles

The functional scope of MNs is greatly expanded by their integration with polymeric microparticles and nanoparticles. The material, fabrication method, and drug-loading technique for these carriers directly determine the drug release kinetics and therapeutic efficacy of the overall system [99]. PLGA remains the foremost choice for the construction of long-acting release systems. Its fabrication is well-established: the water-in-oil-in-water (W/O/W) double emulsion–solvent evaporation method is standard for encapsulating hydrophilic drugs (e.g., proteins, peptides), achieving high encapsulation efficiency without compromising bioactivity, as demonstrated for interleukin-4 (IL-4) and ovalbumin (OVA) [100,101]. For lipophilic or small-molecule drugs (e.g., paclitaxel (PTX), ibuprofen (IBU), and alfacalcidol (ALF)), the oil-in-water (O/W) single emulsion–solvent evaporation method is effective [21,26]. This process involves emulsifying an oil phase (PLGA in dichloromethane or ethyl acetate) into an aqueous phase (containing an emulsifier like PVA), followed by solvent removal. The degradation rate of PLGA, and thus the drug release period (from weeks to months), can be precisely controlled by adjusting the lactic acid to glycolic acid (LA:GA) ratio and the end-group chemistry. For instance, Xu et al. reported that encapsulating ALF-PLGA microparticles within an ethyl cellulose (EC) shell in a core–shell MN extended the drug release to 14 days, enabling bi-weekly administration for osteoporosis [102]. Similarly, high drug-loading PLGA microparticles in a bilayer-dissolving MN provided sustained blood glucose control for over 2 weeks in db/db mice [103]. For applications requiring higher cellular uptake, nanoscale PLGA particles are employed. Their preparation is similar but involves more intense sonication or high-pressure homogenization to achieve more petite sizes. Permana et al. developed PLGA nanoparticles (NPs) loaded with rivastigmine (RV) for brain delivery, with a particle size of approximately 190 nm. NPs with a size ≤ 200 nm demonstrate enhanced brain penetration and longer circulation time compared to those exceeding 200 nm, facilitating passive diffusion into the rat brain. These NPs exhibited controlled drug release over approximately 14 days in vitro. Furthermore, Permana noted that the particle size of the NPs during the formulation process is influenced by several factors, including PLGA concentration, homogenizer speed, and the duration of the secondary homogenization step [104]. Notably, Nguyen et al. systematically compared eight different PLGA microsphere formulations loaded with methotrexate (MTX). Their investigation into the effect of the LA/GA ratio on particle size revealed that a 50/50 ratio with an average molecular weight of 60 kDa yielded the smallest particle size (~897 nm), while a 95/5 ratio with a molecular weight of 40 kDa resulted in a size of 1500 nm. All other tested compositions—50/50 (20, 50, 70, 80 kDa), 10/90 (150 kDa), and 75/25 (50 kDa)—exhibited particle sizes exceeding 2000 nm. The authors noted no significant correlation between in vitro drug release and particle size. The drug release flux was lowest for the PLGA 50/50, 80 kDa formulation (1.98 ± 0.14 μg/h) and highest for the PLGA 50/50, 60 kDa formulation (7.65 ± 0.74 μg/h). The cumulative drug release increased in the following order: 50/50, 80 kDa < 50/50, 20 kDa < 50/50, 70 kDa < 10/90, 150 kDa < 50/50, 50 kDa < 95/5, 40 kDa < 75/25, 50 kDa < 50/50, 60 kDa [105].

In addition to PLGA, there are various functionalized polymers for specific needs. Methoxy poly(ethylene glycol)-poly(ε-caprolactone) (MPEG-PCL) is an amphiphilic block copolymer. Encapsulating hydrophobic drugs with PCL can also be biodegradable. PEG endows the “stealth” property to prolong the circulation time. Hao et al. used the solvent evaporation method to simultaneously load 5-fluorouracil (5-Fu) and indocyanine green (ICG) into MPEG-PCL nanoparticles and prepared a near-infrared light-responsive combination therapy system for the treatment of skin cancer [106]. The Peng team fabricated bifunctional PLGA nanoparticles co-loaded with vitamin E TPGS and hyaluronic acid (HA). TPGS serves as an efficient emulsifier that can target mitochondria to enhance the cytotoxicity of PTX, while the outer HA shell actively targets tumor cells via CD44 receptors. This design allows the MNs to deliver both PTX and ICG and, through polymer degradation, enables continuous release for over six months [26].

**Figure 8:**
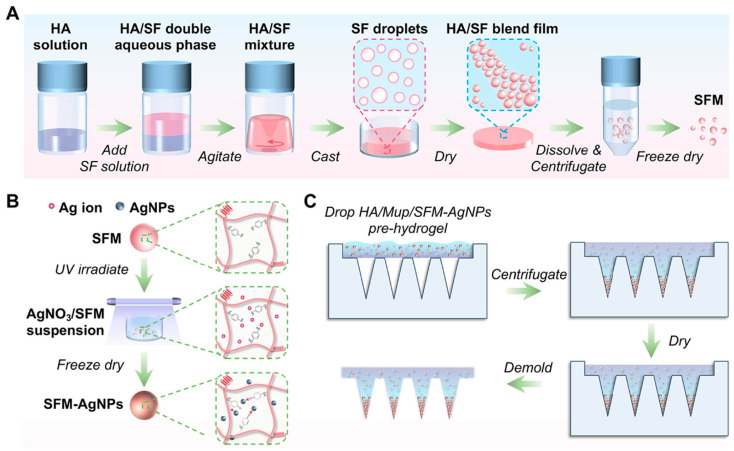
Fabrication of SF microspheres and their integration into an HA-based MN patch. (**A**) Process for fabricating SF microspheres; (**B**) Schematic illustrating the loading of Ag nanoparticles into the microspheres; (**C**) SF microspheres loaded onto a hyaluronic acid (HA) MN platform. Reproduced with permission from Li et al. (2024) [108] © 2024 Elsevier B.V.

Natural polymer SF has great potential. In one application, SF microspheres loaded with levonorgestrel are prepared by a mild aqueous phase method, and this system can achieve sustained release for up to 100 days and can be applied to long-acting contraception [107]. Separately, leveraging the difference in solubility between hyaluronic acid (HA) and SF, Li Yao et al. developed Ag nanoparticle-loaded SF microspheres via thermally induced phase separation. The microspheres are placed in the MN patch together with antibiotics (Figure 8). In mouse models, this composite MN can clear bacterial infections and especially destroy biofilms [108]. Smart and environmentally responsive materials are also being integrated. Li et al. used PCL microspheres to load doxorubicin (Dox) in bacteria-responsive MNs to treat chronic wounds; the microspheres are degraded by lipase upregulated at the infection site, thus realizing targeted antibiotic release within 7 days in vitro [109]. Hu et al. developed acid-responsive star-shaped polylactic-co-glycolic acid copolymer nanoparticles with CaCO_3_ as the environmental response substance. The nanoparticles rapidly degrade in the acidic environment of arthritis, enabling the anti-inflammatory drug Tet to be released rapidly [110].

More complex carrier structures have been engineered for spatiotemporal control and combination therapy. Zheng et al. used the hydrophilic/hydrophobic separation method in the dual-drug MNs to treat skin infections: dissolving the hydrophilic drug polymyxin B in the PVP matrix to achieve rapid release, encapsulating the hydrophobic drug curcumin into PLGA nanoparticles, and then coating the surface of the MNs by electrospray to achieve sustained release and synergistically eliminate biofilms [111]. Bowen Zheng et al. applied a bionic strategy, encapsulating BDNF into PLGA nanoparticles and then wrapping them with macrophage membranes. This coating endows it with immune escape and inflammation site targeting and increases the accumulation of drugs at the lesion in the spinal cord injury model [112]. For intradermal drug reservoirs, there are advantages in combining microspheres and hydrogels. Shi et al. embedded MTX-loaded PLGA microspheres into photocrosslinked gelatin methacryloyl (GelMA) hydrogels to make MN tips. After application, the carrier dissolves, and the GelMA tip containing microspheres is used for MTX release in the skin for 12 days [113]. Yang et al. reported that “missile” MNs are used for wound debridement and healing. The upper part has dextran-coated Ag NPs for deep tissue penetration and mediating lectin-targeted biofilms, and the lower part has porous PLGA microspheres coated with taurine-coated heparin. This system makes the taurine-related immune response last for more than 2 weeks, promoting inflammation resolution and tissue repair [114].

### 3.6. Liposomes

**Figure 9:**
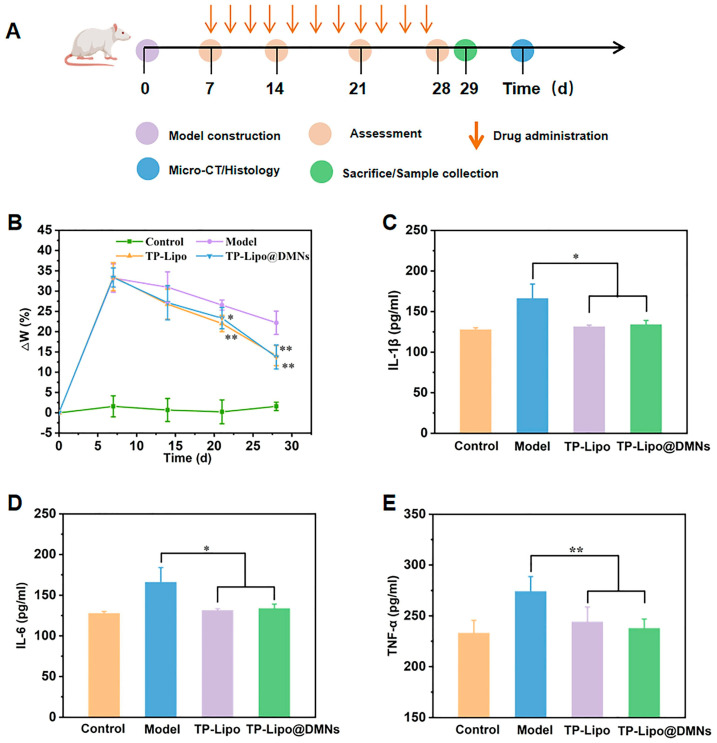
In vivo treatment of osteoarthritis in a rat model. (**A**) A schematic diagram illustrating the establishment of the rat model using sodium mono-iodoacetate (MIA) and the subsequent treatment protocol; (**B**) Representative images of knee joints from the different treatment groups (*n* = 5). Statistical significance compared to the model group is indicated by ** *p* < 0.01; (**C**) Measurements of interleukin-1β (IL-1β) levels (*n* = 4). A significant difference from the model group is indicated by * *p* < 0.05; (**D**) Measurements of interleukin-6 (IL-6) levels (*n* = 4; * *p* < 0.05 vs. model group); (**E**) Measurements of tumor necrosis factor-alpha (TNF-α) levels in serum. Reproduced with permission from Zhou et al. (2021) [119] © 2021 Elsevier B.V.

The integration of liposomes with MN technology has emerged as a key strategy to enhance the efficiency of transdermal drug delivery [115]. Film hydration (Bangham method) is the core preparation method. For example, Alqarni et al. used this method to prepare nano-liposomes containing Enicostemma littorale extract. Their particle size was 91.09 nm, and the polydispersity index was 0.267, with encapsulation efficiency of 82.45%. When using dissolving MNs for drug delivery, the bioavailability was improved [116]. In a study of docetaxel-loaded elastic liposomes, Qiu et al. found that MN pretreatment of the skin can make the transdermal permeation flux reach 1.3–1. A dose of 4 micrograms per square centimeter per hour can reduce the lag time by about 70% and also enhance the delivery effect [117]. The ethanol injection method has also proven advantageous. The encapsulation efficiency of triptolide-loaded liposomes exceeds 90%, and continuous release can be achieved by relying on the MN array [118]. For instance, Zhou and colleagues prepared triptolide-loaded liposomes (TP-Lipo) via ethanol injection for the treatment of osteoarthritis. In vitro release studies revealed that TP-Lipo released approximately 60% of its payload over 24 h, whereas free triptolide (TP) released over 95% within 10 h, demonstrating the liposomes’ sustained-release characteristics. Subsequently, the therapeutic efficacy was evaluated in a monosodium iodoacetate (MIA)-induced rat model of osteoarthritis (Figure 9A). As shown in Figure 9B, knee joint swelling was significantly reduced in rats treated with TP-Lipo and TP-Lipo incorporated into dissolving microneedles (TP-Lipo@DMNs) compared to the model group. Furthermore, the levels of key inflammatory cytokines (TNF-α, IL-1β, IL-6) were measured (Figure 9C–E). The results indicated that the concentrations of these cytokines were substantially lower in the TP-Lipo and TP-Lipo@DMNs groups than in the model group, underscoring the therapeutic potential of the TP-Lipo@DMNs system for managing osteoarthritis [119].

To further optimize release profiles and targeting, liposomes have been functionally modified. Xie et al. prepared flurbiprofen axetil pH-responsive liposomes (with an entrapment efficiency of 75%) by the film hydration-ultrasound method and relied on MN to realize intelligent drug release in the acidic microenvironment at the inflammatory site to reduce joint swelling [120]. Lei et al. reported RGD-peptide-modified liposomes that enhanced cellular uptake via targeting; combined with hyaluronic acid MNs, they achieved simultaneous photodynamic therapy and pain control [121]. The biphasic liposomes prepared by Yang et al. contain 5-aminosalicylic acid and ginsenosides for the treatment of psoriasis. Liposomes can activate the antioxidant pathway. After delivery through shield-shaped hydrogel MNs, the MNs penetrate the thickened stratum corneum, restore oxidative stability and inhibit the expression of IL-17A [122]. Ramalheiro et al. prepared phytantriol-based cubosome-like nanoparticles via a solvent exchange method. These particles achieved a high encapsulation efficiency of 94.6%, with an average diameter of 221 ± 14 nm and a polydispersity index ranging from 0.2 to 0.4. When incorporated into fast-dissolving microneedles, the nanoparticles enabled sustained release of rapamycin over 14 days. This work underscores the potential of lipid-based nanoparticle systems for long-acting therapeutic applications [123]. Qu et al. prepared a series of anionic and cationic liposomes loaded with either ICG or dexamethasone using the thin-film hydration and extrusion method. The anionic liposomes, composed of DPPC, DSPE-mPEG2000, and cholesterol at a molar ratio of 55:5:40, were extruded through polycarbonate membranes with pore sizes of 100, 200, and 400 nm. This process yielded particles with mean diameters of 91.56 ± 2.51 nm (termed 100 nm-lip), 260.70 ± 11.06 nm (250 nm-lip), and 461.53 ± 8.97 nm (450 nm-lip). These anionic liposomes exhibited a zeta potential of approximately –3.5 mV and demonstrated good stability. For the cationic liposomes, surface charge was precisely modulated by systematically varying the molar percentage of the cationic lipid DOTAP (20%, 40%, 60%), while maintaining a fixed cholesterol content of 20% and using DOPE to complete the formulation (corresponding molar ratios: 20:60:20, 40:40:20, 60:20:20). The resulting particles had uniform sizes (approximately 255–260 nm), and their zeta potential increased significantly with higher DOTAP content, rising from 23.17 ± 0.84 mV for the 20% group to 32.17 ± 0.38 mV for the 60% group. The study revealed that while the 100 nm anionic liposomes showed high cellular uptake in vitro, the 250 nm variant offered the best balance between skin retention and cellular delivery. Furthermore, converting the liposomes to a strongly cationic state (e.g., the 60% DOTAP group) via increased DOTAP content significantly prolonged skin retention time to 132 h and greatly enhanced cellular uptake efficiency, primarily due to stronger electrostatic interactions [124]. The loaded liposome MNs are currently made by dissolving materials such as polyvinylpyrrolidone and hyaluronic acid and then molding or photocuring. The MNs create microchannels for liposomes, but the drug release rate and specificity mainly depend on the composition, surface condition, and response of the liposomes [125,126].

## 4. The Clinical Translation Landscape of Microneedles

Despite their broad therapeutic potential, the clinical translation of microneedles (MNs) is still in its early stages. Hollow MNs, which enable precise fluid injection, are currently the most widely used type in clinical practice. Approved examples, such as the BD Soluvia™ and MicronJet 600™, are used for the intradermal delivery of drugs, including influenza vaccines and insulin [127]. By mechanically creating a channel for quantitative liquid infusion, this technology has amassed substantial clinical data in areas like vaccination and diabetes management. Its translational advantages lie in high technical maturity, precise dose control, and compatibility with conventional clinical practices [128]. The clinical translation of radiofrequency MNs and MN rollers remains more niche. Radiofrequency MNs, which combine thermal effects with drug delivery, are primarily used in aesthetic dermatology for applications like scar revision and skin tightening. While several commercial products exist, the supporting clinical evidence is often fragmented. MN rollers, among the earliest commercialized types, are used for minimally invasive skin treatments. However, inconsistencies in manual operation lead to variable clinical data, limiting their role largely to adjunctive therapy [129].

In contrast, the clinical translation of dissolving/degradable MNs remains preliminary, with most progress concentrated on improving pharmacology and pharmacokinetics (PK) in preclinical models. Screening in small animal models (e.g., mice, rats) is typically used for initial PK and pharmacodynamic (PD) evaluations [130]. These MNs use natural (e.g., hyaluronic acid, chitosan) or synthetic degradable polymers (e.g., PLGA, PVA) as carriers. Drugs are embedded within the matrix, and upon skin insertion, the needles dissolve or degrade via body fluids to release the drug in a sustained manner. This eliminates needle retrieval, enhancing safety [131]. However, several bottlenecks hinder their translation. First, material properties often mismatch clinical needs. Current materials lack the tunable mechanical strength required for different tissues; for instance, penetrating highly keratinized gingival tissue demands greater strength, while application on dynamic oral mucosa requires flexibility. No single material currently meets all such requirements [132]. Second, controlling drug loading and release is challenging. Large-molecule drugs (e.g., proteins, nucleic acids) can be destabilized during fabrication, and precisely modulating their release profile is difficult, leading to inconsistent therapeutic outcomes. Finally, scalable manufacturing and quality control are problematic. Precisely controlling needle dimensions and ensuring uniform drug loading across batches with current processes results in low yield, impeding commercial scale-up.

Accelerating the translation of dissolving/degradable MNs requires a coordinated effort across four dimensions: material optimization, process engineering, clinical trial design, and regulatory adaptation. Material optimization is central, necessitating the development of composite matrices that balance mechanical strength, biocompatibility, and controlled degradation. Process engineering is key to scalable production. Current methods like template casting are inefficient and yield high batch-to-batch variability, calling for the development of high-precision, high-throughput fabrication technologies. Concurrently, rigorous quality control standards must be established to quantify critical parameters (e.g., needle dimension, drug loading, degradation rate) to ensure consistency and safety in clinical use [133]. Prior to human trials, systematic PK/PD studies in large animal models with relevant skin structure (e.g., minipigs, non-human primates) are essential to better predict delivery efficiency, duration of action, and potential toxicity in humans. Furthermore, clinical trial design must reflect real-world use. Expanding sample sizes to include diverse populations across age, gender, and disease severity will validate generalizability. Endpoint design should be optimized to include patient-reported outcomes (e.g., pain scores, ease of use) alongside traditional efficacy measures, enhancing clinical acceptance. Dedicated trials for special populations (e.g., children, the elderly, immunocompromised individuals) are also crucial. For instance, pediatric vaccine MN trials must specifically assess safety and tolerability, employing MN dimensions (e.g., length ≤ 500 μm) and application methods suited for children. Regulatory adaptation is a vital enabler. Currently classified as drug-device combination products, dissolving/degradable MNs face dual regulatory complexity. A dedicated framework is needed to clarify classification, registration pathways, and technical requirements. Specific quality standards and clinical evaluation guidelines should be established for critical aspects like material biocompatibility, drug stability, and degradation product safety. Concurrently, promoting industry-wide standards to unify terminology, performance testing methods, and clinical endpoints is necessary to ensure consistency.

Looking forward, advances in materials science, manufacturing, and clinical research will likely enable the translation of dissolving/degradable MNs into broader applications, such as long-acting therapy for chronic diseases and integrated biosensing-and-treatment systems. The development of personalized MNs will also emerge as a key research frontier. Although significant translational challenges remain, the gradual resolution of these technical bottlenecks will establish dissolving/degradable MNs as important tools in precision medicine, offering safer, more efficient, and patient-friendly therapeutic solutions.

## 5. Conclusions and Future Prospects

MN technology has rapidly evolved over the past two decades into a promising drug delivery platform, positioning it as a viable alternative to conventional injections and oral administration. The microneedle array penetrates the barrier of the outer layer of the skin to achieve efficient drug delivery. In animal models and early clinical trials, studies show that the insertion rate is high in pig and rodent models, the bioavailability is better than traditional patches, and it also has high patient acceptance because it is painless and can be self-administered.

Long-acting MNs are different from fast-release microneedles. Its design is to make the drug release continuously. To keep the blood drug concentration stable, minimize the peak-trough fluctuations, enhance the curative effect, reduce side effects, and replace multiple administrations with a single dose. At present, the research on long-acting MNs mainly focuses on biodegradable or swellable polymers. Synthetic polymers such as polylactic-co-glycolic acid (PLGA) and polylactic acid (PLA) are widely used because their degradation rate can be adjusted and the release time can be set from several weeks to several months. Natural polymers such as chitosan (CS), silk fibroin (SF), and hyaluronic acid (HA) are widely used in immunotherapy and anti-infection because of their good biocompatibility and mechanical strength.

Integrating drug-loaded nanoparticles, such as liposomes, microspheres, etc., can further prolong the release time and improve the drug-loading capacity. Current research shows that long-acting microneedles can deliver a variety of drugs, including hydrophobic, hydrophilic, macromolecular, small molecular, etc., and are feasible, but there are still many deficiencies in comprehensive clinical research. There is an urgent need for more in vivo efficacy and long-term toxicity studies. In addition, we need to solve the production problems of batch consistency and aseptic control. However, with the accumulation of clinical data, long-acting microneedle preparations may become a model-changing approach in chronic disease management.

## Data Availability

The data presented in this study are available on request from the corresponding author.
